# Renal tissue microbiota and metabolite profiling reveal dysregulated signatures in diabetic kidney disease

**DOI:** 10.3389/fmicb.2026.1898177

**Published:** 2026-09-17

**Authors:** Na Zhao, Zijia Leng, Qiqi Li, Shan Wei, Meng Li, Yu Zhang, Chen Wang

**Affiliations:** 1 The Basic Medical College of Shanxi Medical University, Taiyuan, Shanxi, China; 2 Second Clinical Medical College of Shanxi Medical University, Taiyuan, Shanxi, China; 3 Key Laboratory of Cellular Physiology, Ministry of Education, Department of Physiology, Shanxi Medical University, Taiyuan, China; 4 Shanxi Provincial Key Laboratory of Aging-related Diseases Mechanisms and Translation, Taiyuan, China

**Keywords:** biomarkers, diabetic kidney disease, metabolomics, pathological features, renal microbiome

## Abstract

**Introduction:**

Diabetic kidney disease (DKD) has become the main cause of end-stage renal disease in China. Mounting evidence links microecological disorders to DKD progression. However, the composition and the functional characteristics of the renal microecology in DKD patients remain poorly defined. This study characterized renal tissue microbiota and metabolite profiles in DKD patients, aiming to provide insights for novel diagnostic strategies for DKD.

**Methods:**

Renal tissue microbiome was analyzed in 42 DKD patients and 10 controls via 16S ribosomal DNA sequencing. Renal metabolomics was performed in 20 DKD patients and 10 controls using liquid chromatography-tandem mass spectrometry (LC-MS/MS). Spearman correlation analysis clarified associations of clinical parameters with renal microbiome and metabolites. Biomarkers were identified by multi-omics integration.

**Results:**

IHC, IF and TEM reveals the presence of bacteria in both glomeruli and renal tubules. A significant separation in microbial community composition was observed between DKD patients and controls (*p* < 0.05). The relative abundance of *Acidobacteriota*, *Acinetobacter* and *Afipia* genus were significantly elevated in DKD group, whereas *Ralstonia* genus was decreased. Notably, the genus *Acinetobacter* demonstrated a significant negative correlation with the eGFR, while *Afipia* genus exhibited a significant positive correlation with blood glucose levels. Renal microbiota resembled urinary microbiota more closely than gut microbiota. Metabolomic analysis revealed significant difference in renal tissue metabolites between DKD patients and healthy controls. Lactate, hypoxanthine and phosphorylcholine were significantly positively correlated with the eGFR. Comprehensive multivariate analyses identified the genus *Ralstonia* as a crucial biomarker associated with renal fibrotic injury in DKD, consistent across urinary and gut microbiomes. Decreased serum levels of stearylcarnitine (Car18:0), oleylcarnitine (Car18:1) and tryptophan were closely associated with renal injury, whereas decreased urinary serine and tyramine levels reflected the change of renal metabolites in DKD.

**Conclusion:**

Our finding confirms the existence of microbiota within renal tissue and demonstrates that its structure and composition are significantly altered in DKD. These disruptions in renal microecology are closely associated with the progression of DKD. Multi-omics analysis further identified a panel of candidate markers for evaluation of DKDs.

## 1 Introduction

DKD is one of the most common complications of type 2 diabetes which is a global public health issue (Locatelli et al., 2004). It is about 39 million diabetic patients with chronic kidney disease, including 36.9 million patients with proteinuria and 6.6 million patients with reduced estimated glomerular filtration rate (eGFR) in China (Jia et al., 2025). DKD has become the main cause of chronic kidney disease and end-stage renal disease in China, and it is strongly associated with poor clinical outcomes, including severe renal failure, uremia and increased mortlity (Xu et al., 2024).

The human microbiota is a normal constituent of the human ecosystem, with the majority residing in the colon. Intestinal microbiota performs unique metabolic functions absent in the human host, such as digesting complex polysaccharides, synthesizing short-chain fatty acids, regulating host metabolism and promoting immune system maturation (Bäckhed et al., 2005; Zhao et al., 2018; Visconti et al., 2019; Fan and Pedersen, 2021; Donald and Finlay, 2023; Panwar et al., 2023; Keung et al., 2025). Beyond the gut, microbiota has also been identified in adipose tissue, lung tissue, breast tissue, renal tissue and the urinary system (Whiteside et al., 2015; Hieken et al., 2016; Massier et al., 2020; Wang et al., 2022; Li et al., 2024). Wang *et.al* reported that metabolites derived from commensal microbiota in triple-negative breast cancer were associated with enhanced efficacy of immunotherapy (Wang et al., 2022). Lucas *et.al* indicated that bacteria within adipose tissue play an important role in initiating and maintaining subclinical adipose tissue inflammation, thereby influencing the metabolic sequelae of obesity demonstrated that renal bacterial function was linked to hypertension (Liu et al., 2022). Collectively, these findings indicate that tissue and organ-resident microbiota and their metabolites may modulate disease initiation and progression. Although numerous studies have demonstrated the presence of bacteria in renal tissue, the crosstalk between commensal microbiota and the kidney in DKD remains poorly understood due to limited clinical cohorts and mechanistic investigations.

In this study, we performed microbiome profiling on renal tissues from 42 patients with DKD and 10 healthy controls, and conducted metabolomic analysis on renal tissues from 20 DKD patients and 10 healthy controls. Based on our analysis, the genus *Ralstonia* exhibited a close association with DKD. Interestingly, stearylcarnitine (Car18:0) and oleylcarnitine (Car18:1) were significantly correlated with the eGFR, a key indicator for assessing DKD severity. These findings suggest that the renal microbiota and metabolism profiles may serve as promising biomarker for DKD detection and potential therapeutic targets.

## 2 Materials and methods

### 2.1 Study participants

This retrospective observational study included patients who underwent renal biopsy and were diagnosed with diabetic kidney disease (DKD) at the Second Hospital of Shanxi Medical University between June 2021 and June 2024. DKD was diagnosed according to the Renal Pathology Society (RPS) classification (Tervaert et al., 2010).

The 16S rDNA sequencing cohort included 42 DKD patients and 10 controls, and the metabolomics cohort included 20 DKD patients and 10 controls. Control renal tissues were obtained from non-neoplastic parenchyma located more than 5 cm away from the tumor margin, harvested from radical nephrectomy specimens of patients with renal cell carcinoma who had no history of diabetes mellitus or other renal disorders, and histological examination verified their normal morphology. Percutaneous renal biopsies were performed under ultrasound guidance using a 16-gauge automated spring-loaded biopsy needle (Bard Magnum, Bard Biopsy Systems, USA) by a nephrologist, following the protocol described previously (Liu et al., 2022). All biopsy specimens were independently scored by two experienced pathologists who were blinded to clinical and demographic data; any discrepancies were resolved by re-review to achieve consensus. The sample inclusion and exclusion criteria are detailed in the study flow diagram (Supplementary Figure 1). This study was approved by the Institutional Ethics Committee of Second Hospital of Shanxi Medical University. Data collection and analysis were conducted in accordance with the requirements of the National Health Commission of the People’s Republic of China. Written informed consent was obtained from all participants.

All renal tissues were immediately collected in the operating room, snap-frozen in liquid nitrogen, and stored at −80°C until further processing, without undergoing any freeze-thaw cycles prior to analysis.

### 2.2 16 S rDNA sequencing and analysis

Microbial DNA was extracted from renal tissue samples using the E.Z.N.A.^®^ Tissue DNA Kit (Omega Bio-tek, Norcross, GA, U.S.) according to the manufacturer’s protocols. The V3–V4 region of the bacterial 16S ribosomal RNA gene was amplified by PCR using the forward primer 341F (5’-CCTAYGGGRBGCASCAG-3’) and the reverse primer 806R (5’-GGACTACNNGGGTWTCTAAT-3’), where each primer contained an eight-base barcode unique to each sample. PCR reactions were performed in triplicate in 20 μL mixtures containing 4 μL of 5 × FastPfu Buffer, 2 μL of 2.5 mM dNTPs, 0.8 μL of each primer (5 μM), 0.4 μL of FastPfu Polymerase, and 10 ng of template DNA. The cycling conditions were: initial denaturation at 95 °C for 2 min, followed by 27 cycles of denaturation at 95 °C for 30 s, annealing at 55 °C for 30 s, and extension at 72 °C for 60 s, with a final extension at 72 °C for 5 min. Amplicons were extracted from 2% agarose gels and purified using the AxyPrep DNA Gel Extraction Kit (Axygen Biosciences, Union City, CA, U.S.) according to the manufacturer’s instructions.

Purified amplicons were pooled in equimolar concentrations and paired-end sequenced (2 × 250 bp) on an Illumina NovaSeq 6000 platform by Shanghai Biozeron Biotechnology Co., Ltd., (Shanghai, China). The sequencing run generated a total of 2,330,692 raw reads across all samples, with a mean of 44,821 reads per sample (based on 52 samples). The read length distribution revealed that 99.93% of reads fell within the 401–500 bp range, confirming successful amplification of the V3–V4 region.

Raw paired-end reads were demultiplexed and quality-filtered. Low-quality reads were trimmed using Trimmomatic with a sliding window quality threshold of Q20. High-quality reads were then clustered into operational taxonomic units (OTUs) at 97% similarity using UPARSE (version 7.1). Chimeric sequences were identified and removed using UCHIME. Taxonomic assignment of OTUs was performed against the SILVA database (version 138) using the RDP Classifier with a confidence threshold of 70%.

Given the low-biomass nature of renal tissue samples, we applied a multi-strategy filtering approach to minimize the impact of potential contaminants. First, following the filtering strategy described by Borchmann (2021), we excluded (i) taxa frequently detected in negative controls, (ii) taxa detected in only very few samples, and (iii) taxa that have been previously described as sequencing contaminants. Second, we adopted a knowledge-based contaminant removal approach informed by external negative controls, as recently applied in a renal microbiome study published in Nature Communications (Agudelo et al., 2024). After stringent filtering, low-confidence and suspected contaminant taxa were excluded from downstream analyses.

### 2.3 Metabolite extraction

For metabolite extraction, approximately 25 mg of frozen renal tissue was weighed into a 2 mL Eppendorf tube containing two homogenization beads. Then, 500 μL of extraction solvent [methanol:acetonitrile:water = 2:2:1 (v/v/v)] spiked with deuterated internal standards was added. The mixture was vortexed for 30 s, followed by homogenization at 35 Hz for 4 min using a tissue homogenizer, and then subjected to ultrasonication in an ice-water bath for 5 min. This homogenization-ultrasonication cycle was repeated three times. The samples were then incubated at −40 °C for 1 h to precipitate proteins, followed by centrifugation at 12,000 rpm (13,800 × *g*, radius 8.6 cm) for 15 min at 4°C. The resulting supernatants were transferred to autosampler vials for LC-MS/MS analysis. Additionally, an equal volume of supernatant from each sample was pooled to prepare quality control (QC) samples, which were injected periodically throughout the analytical run to monitor system stability and reproducibility.

### 2.4 LC-MS/MS analysis

Chromatographic separation was performed on a Vanquish UHPLC system (Thermo Fisher Scientific) equipped with a Waters ACQUITY UPLC BEH Amide column (2.1 mm × 50 mm, 1.7 μm). The mobile phase consisted of phase A: 25 mmol/L ammonium acetate and 25 mmol/L ammonium hydroxide in water (pH 9.75) and phase B: acetonitrile. The autosampler temperature was maintained at 4 °C, and the injection volume was 2 μL.

Mass spectrometry detection was carried out on an Orbitrap Exploris 120 mass spectrometer (Thermo Fisher Scientific) controlled by Xcalibur software (version 4.4), operating in information-dependent acquisition (IDA) mode. The ESI source parameters were set as follows: sheath gas flow rate 50 Arb, auxiliary gas flow rate 15 Arb, capillary temperature 320°C, full MS resolution 60,000, MS/MS resolution 15,000, stepped normalized collision energy (sNCE) 20/30/40, and spray voltage 3.8 kV (positive mode)/-3.4 kV (negative mode).

The raw data were converted to the mzXML format using ProteoWizard and processed with an in-house program developed using R and based on XCMS for feature detection, extraction, alignment, and integration. Metabolite identification was performed using the BiotreeDB (V3.0) database. Identification confidence levels were assigned according to the Metabolomics Standards Initiative (MSI) reporting standards (Sumner et al., 2007).

### 2.5 Fluorescence *in situ* hybridization (FISH)

Bacteria within the renal tissue were identified in paraformaldehyde-fixed tissue sections. Paraffin-embedded tissues were deparaffinized and hybridized to a universal bacterial probe (EUB338:5′-GCTGCCTCCCGTAGGAGT-3′). All probes were labeled with the Cy5 fluorophore and obtained from the Exonbio Lab (Guangzhou, China). Hybridization was performed overnight at 56°C, followed by washing and counterstaining with the nuclear dye, 2-(4-Amidinophenyl)-6-indolecarbamidine dihydrochloride (DAPI).

### 2.6 Hematoxylin and eosin (H&E) staining and immunostaining

Deparaffinized sections were subjected to antigen retrieval by high-pressure heating in EDTA buffer for 2.5 min. The sections were then incubated overnight with primary antibodies against LPS, followed by incubation with goat anti-rabbit secondary antibody at room temperature for 50 min. Finally, the sections were counterstained with hematoxylin.

### 2.7 Transmission electron microscopy (TEM)

Renal tissues were pre-fixed in 3% glutaraldehyde for 12 h, followed by post-fixation in 3% osmium tetroxide at 4 ° for 90 min. After dehydration in a graded acetone series, the specimens were embedded in epoxy resin. Ultrathin sections (50 nm) were prepared and stained with uranyl acetate and lead citrate at room temperature for 15–20 min, and subsequently observed and imaged under TEM.

### 2.8 Statistical analysis

Data analyses were performed using R software (version 4.0.2). Data were standardized and log-transformed to reduce noise and mitigate the effects of high variability. Following transformation, alpha diversity was used to measure microbial species richness within individual samples. Beta diversity of the microbiota was assessed using principal coordinates analysis (PCoA). PCoA was used to visualize differences in microbial community structure between samples. Bray-Curtis distance was employed to calculate compositional dissimilarities between samples. Between-group differences were assessed using two independent methods: PERMANOVA (adonis2 function, 999 permutations) and MRPP (mrpp function, 999 permutations). PERMDISP (betadisper function, 999 permutations) was additionally performed to test for homogeneity of multivariate dispersions among groups. 95% confidence ellipses were added to the PCoA plot to visually display the clustering pattern of samples within each group. Statistically candidate differential features between groups were identified through LEfSe analysis and random forest prediction model. To evaluate the discriminatory ability of the random forest model based on genus-level microbial abundance for distinguishing DKD from controls, we performed 10-fold cross-validation to assess the generalizability of the model and mitigate the risk of overfitting. Model performance was evaluated using the area under the receiver operating characteristic (ROC) curve (AUC) with its 95% confidence interval (CI). The optimal cutoff point was determined by the Youden index, and the corresponding sensitivity and specificity were reported.

Statistical analyses were conducted using SPSS version 26.0. Clinical data of DKD patients and healthy controls are presented as mean ± SD or median, while categorical data are expressed as percentages. The Student’s *t*-test or non-parametric tests were used for comparing continuous variables, and the chi-square test was applied for categorical variable.

## 3 Results

### 3.1 The baseline characteristics of DKD patients and controls

Renal tissue samples from 42 DKD patients and 10 controls were included in a cohort for renal microbiota sequencing and baseline clinical characteristic analysis (Table 1). The eGFR was significantly lower in the DKD group (*p* < 0.001) compared to controls. Biochemical parameters, including blood glucose, serum creatinine and urea nitrogen, exhibited significant differences between the DKD and control groups (*p* < 0.05). No significant differences in age, sex, or body mass index (BMI) were observed between the two groups.

**TABLE 1:** Clinical characteristics of the 16S rDNA sequencing cohort (‘x ± s).

Variables	control (*n* = 10)	DKD (*n* = 42)	*P*
eGFR (ml/min/1.73 m^2^)	110.11 ± 21.60	52.46 ± 31.92	< 0.001
H (cm)	165.80 ± 7.67	167.95 ± 8.03	0.446
W (kg)	65.35 ± 14.16	73.39 ± 11.96	0.071
BMI (kg/m^2^)	23.57 ± 3.55	25.96 ± 3.43	0.055
Age (years)	54.00 (52.25, 64.00)	56.00 (48.25, 62.75)	0.954
Scr (μmol/L)	65.00 (56.00, 83.50)	139.50 (102.75, 242.73)	< 0.001
BUN (mmol/L)	6.30 (5.15, 8.12)	12.40 (8.45, 15.37)	0.002
Hb (g/L)	132.62 (119.25, 132.62)	121.74 (114.00, 130.75)	0.211
Alb (g/L)	43.45 (40.28, 45.17)	33.49 (28.50, 37.50)	< 0.001
GLU (mmol/L)	4.77 (4.31, 5.03)	8.95 (7.75, 8.95)	< 0.001
Sex (Male)	6 (60.00)	31 (73.81)	0.633

Alb, serum albumin; Hb, hemoglobin; eGFR, estimated glomerular filtration rate; GLU, fasting blood glucose; BMI, body mass index; Cr, serum creatinine; SBP, systolic blood pressure; DBP, diastolic blood pressure.

### 3.2 Presence of microbiota in renal tissues of DKD patients

FISH was performed using a universal bacterial probe (EUB338) combined with immunofluorescence to visualize bacteria in renal specimens from DKD patients and controls. Bacteria were detected in renal specimens from both DKD patients and controls (Figure 1A). Immunohistochemical results demonstrated LPS deposition in both DKD and control renal specimens, primarily localized within renal tubules and peritubular interstitium (Figure 1B). Furthermore, bacterial structures were further identified within the cytoplasm of interstitial cells in renal specimens of DKD patients by TEM (Figure 1C).

**FIGURE 1:**
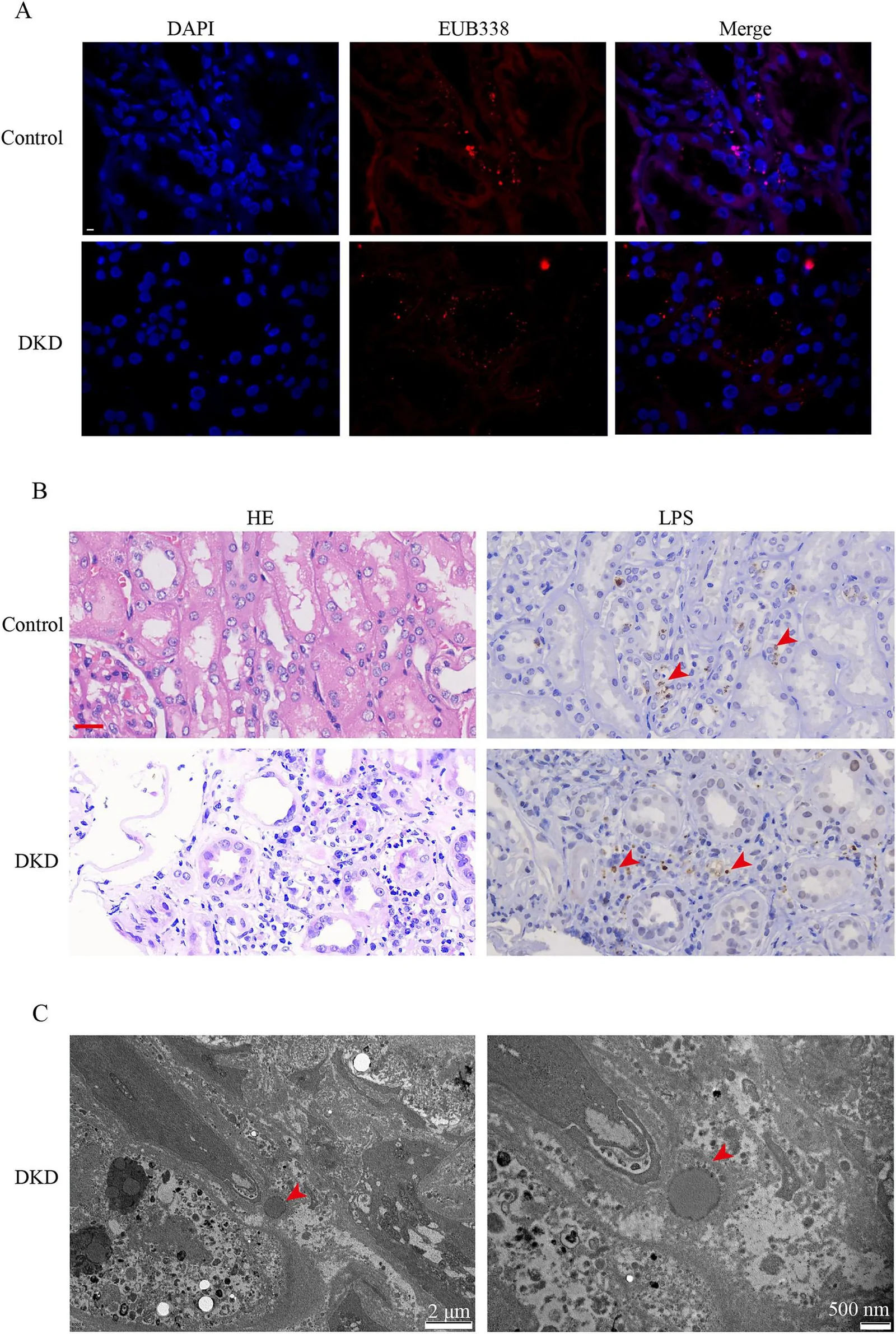
Detection of bacterial presence and localization in renal tissues of DKD patients. **(A)** FISH using the universal bacterial probe EUB338 on a paraffin section of renal tissue from a DKD patient. **(B)** Representative H&E staining and specific antibody staining targeting LPS in renal tissues. Arrows indicate positive staining. **(C)** TEM images of renal specimens from DKD patients show bacterial morphology within cytoplasm. Arrows indicate bacterial structures.

### 3.3 Altered renal microbiome composition in patients with DKD

To investigate the differences in renal tissue characteristics between patients with DKD and controls, renal biopsy specimens from 42 DKD patients and 10 normal controls were analyzed by 16S rDNA gene sequencing. Rarefaction curves, constructed based on the number of sequenced reads and OTU counts, plateaued as sequencing depth, indicating that the sequencing coverage was sufficient to comprehensively represent the microbial diversity in the samples (Figure 2A). Although no statistically significant difference in alpha diversity was observed between the two groups (*p* > 0.05), DKD patients exhibited a trend toward decreased microbial richness compared to controls (Figure 2B). PCoA based on Bray-Curtis distance revealed a significant separation in microbial community composition between the DKD and control groups (Figure 2C). PERMANOVA analysis (999 permutations) confirmed that the grouping factor significantly contributed to the variation in community composition (R^2^ = 0.0827, *P* = 0.006). MRPP analysis further supported the between-group differences (*A* = 0.046, *P* = 0.001). PERMDISP analysis showed no significant difference in within-group dispersion between the two groups (*F* = 0.766, *P* = 0.401), indicating that the observed separation was driven by differences in community centroids rather than differences in within-group variability. The relative abundance of microbial taxa at the genus level was visualized using stacked bar charts for both groups, which illustrated the similarities and differences in their community structures (Figure 2D). The top five most abundant genera were *Acidovorax*, *Acinetobacter*, *Afipia*, *Ralstonia* and *Sediminibacterium*. Wilcoxon rank-sum tests were performed to identify differentially abundant taxa between the two groups at the phylum and genus levels. Compared with controls, DKD patients showed a significantly higher abundance of *Acidovorax*, *Acinetobacter*, *Afipia*, *Sediminibacterium*, whereas the abundance of *Ralstonia* was significantly reduced (Figure 2E). Using external cohort data from previous studies, alterations in core renal microbial species were validated in both urinary and gut microbiota. In the gut microbiome, two core species were shared with the renal microbiota (Figure 2F), while seven core species were shared between the urinary microbiome and the renal microbiota (Figure 2G), indicating that the microbial species in renal tissue were more similar to those in the urinary microbiome.

**FIGURE 2:**
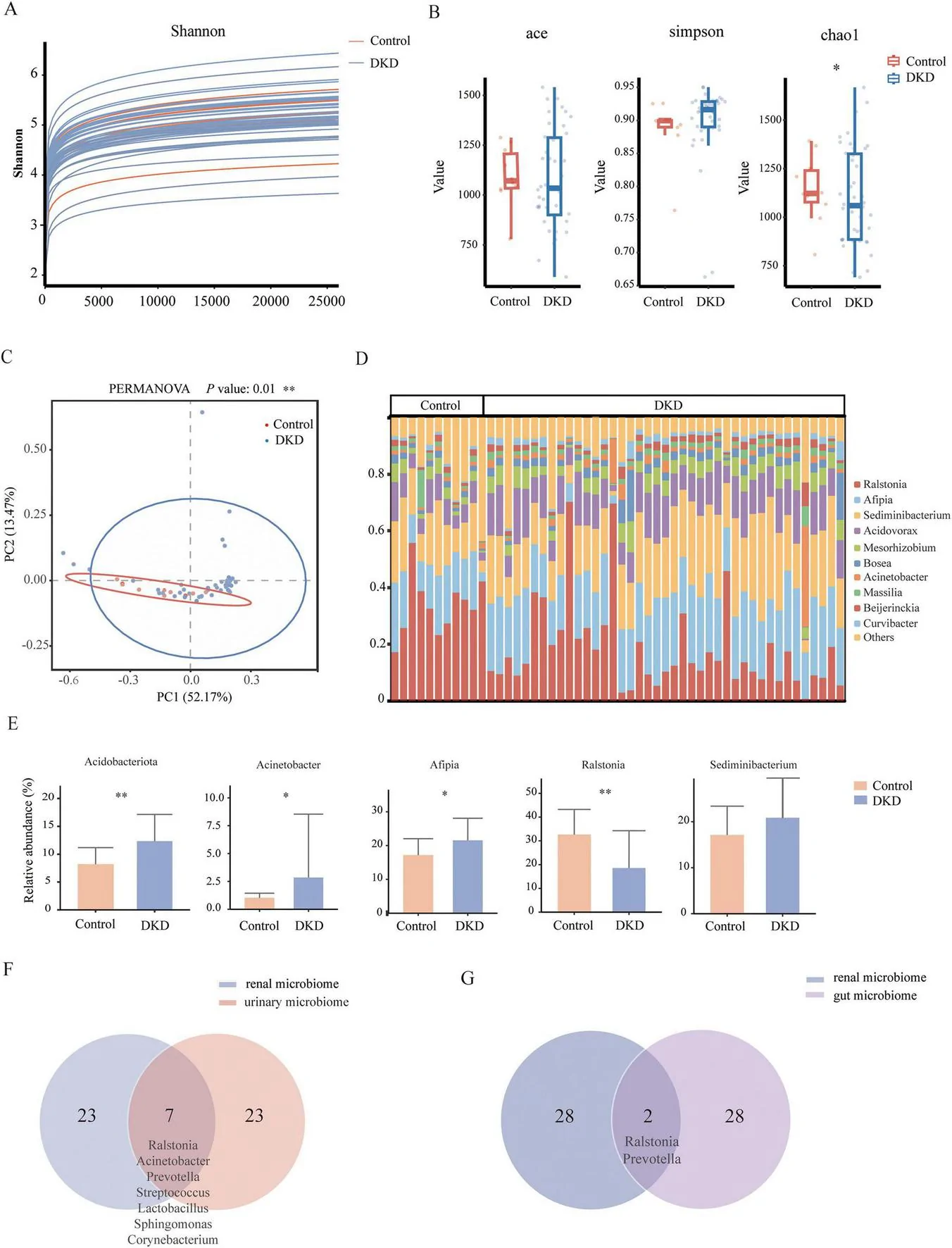
Differences in diversity and composition of renal microbial communities between DKD group and control group. **(A)** Rarefaction curves comparing renal microbial communities between DKD and control groups, showing differences in richness and evenness. The *x*-axis represents the number of sequenced reads, while the *y*-axis indicates the observed OTUs. **(B)** Box plots displaying differences in Ace, Chao1, and Shannon diversity indices of renal microbiota between groups. **(C)** PCoA analysis of renal microbiota. Principal coordinates analysis (PCoA) based on Bray-Curtis distance showing the distribution of DKD and control samples. Ellipses represent 95% confidence intervals. The *x*-axis (PC1) and *y*-axis (PC2) represent the scores of the first and second principal components. Each point represents one sample, with color and shape indicating different groups. **(D)** Stacked bar chart showing relative abundance of renal microbiome at the genus level in DKD and control groups, demonstrating proportional differences in microbial community structure. The *x*-axis represents group samples, while the *y*-axis shows relative abundance of bacterial taxa. **(E)** Comparison of top 5 most abundant genera between groups using Wilcoxon rank-sum test. Each subpanel represents one taxon, with box plots showing distribution in DKD (blue) and control (red) groups. Box plots reveal the most differentially abundant bacterial taxa at genus level. *p < 0.05; **p < 0.01; ***p < 0.001. **(F,G)** Venn diagram showing the screening of the union of the microbiome.

### 3.4 Identification of characteristic microbiota and functional prediction in DKD and control groups

To identify characteristic microbiota in DKD and control groups, the linear discriminate analysis (LDA) effect size (LEfSe) algorithm was employed to screen for microbiota with LDA scores > 3.0 and ranking among the top 15 in abundance (Figures 3A,B). The analysis revealed significant differences in characteristic microbiota between the DKD and control groups across multiple taxonomic levels from phylum to genus. At the genus level, the DKD group exhibited a higher abundance of *Afipia* and *Acidovorax* compared to controls. In contrast, the controls showed a higher relative abundance of *Burkholderia* and *Ralstonia* (Figure 3B).

**FIGURE 3:**
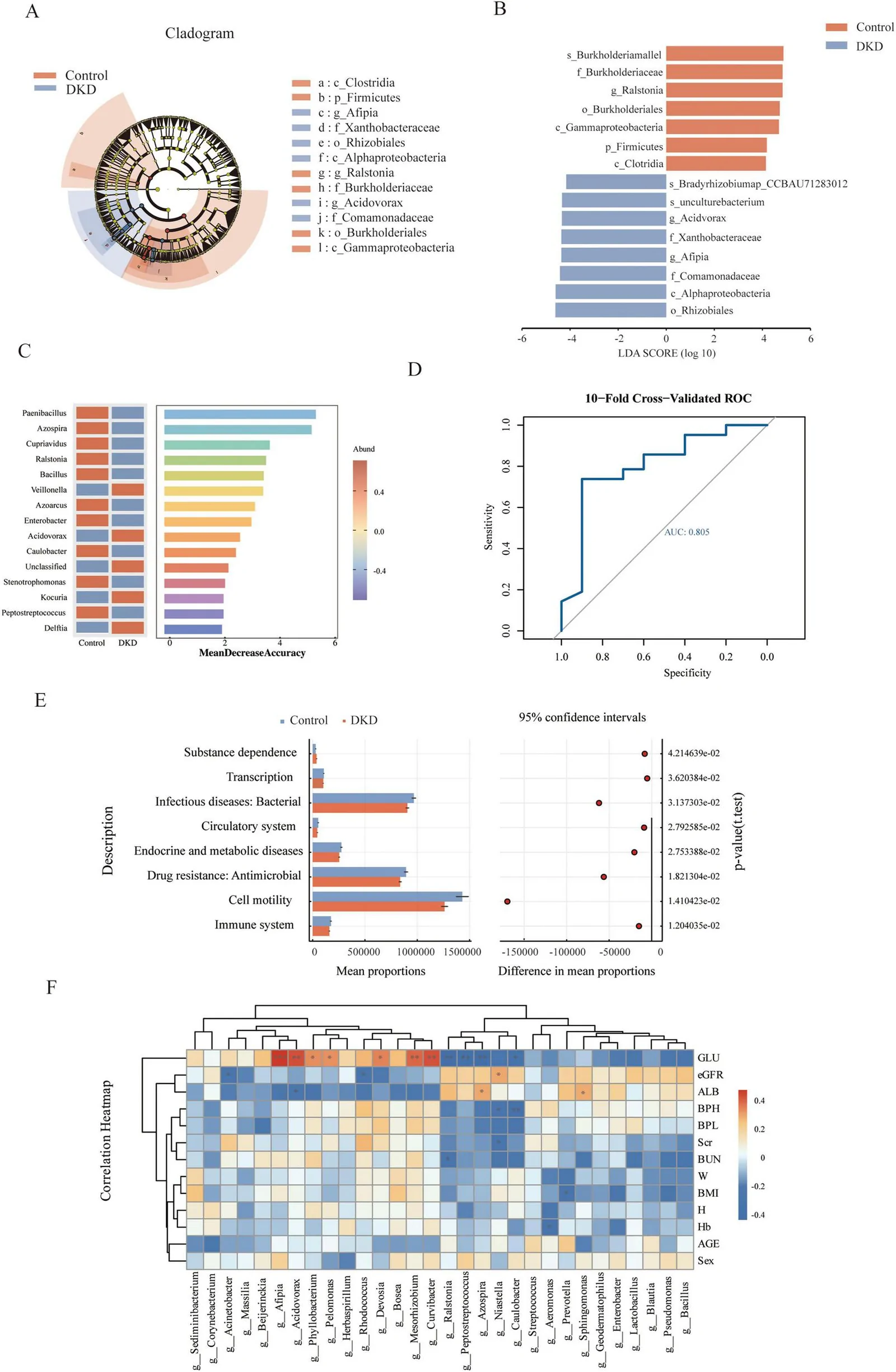
Filtering of characteristic microbiota and analysis of their functions and correlations. **(A,B)** LEfSe analysis identified characteristic microbial functions of different groups, with an LDA score threshold > 3. **(C)** Random forest classification calculating the importance of microbial biomarkers between groups. Left panel shows the abundance of each listed feature in each group using the random forest method; right panel shows feature importance for each group. **(D)** Receiver operating characteristic (ROC) curve of the random forest model based on 10-fold cross-validation. The shaded area represents the 95% confidence interval of the AUC. **(E)** KEGG pathway analysis comparing predicted functions of renal microbiota between DKD and control groups, with statistical significance assessed using Wilcoxon rank-sum test. **(F)** Heatmap showing correlations between differential species in renal microbiota and clinical parameters of DKD at the genus level. Alb, serum albumin; Hb, hemoglobin; eGFR, estimated glomerular filtration rate; GLU, blood glucose; BMI, body mass index; Cr, serum creatinine; SBP, systolic blood pressure; DBP, diastolic blood pressure. Spearman correlation analysis. *p < 0.05; **p < 0.01; ***p < 0.001.

A random forest classification model was constructed to further investigate the potential role of differential microbiota in disease development (Figure 3C). Fifteen characteristic microbiotas with Mean Decrease Accuracy values > 2 were identified between the two groups. Among these, *Paenibacillus, Azospirillum, Cupriavidus, Ralstonia* and *Bacillus* showed decreased abundance in the renal microbiome of DKD patients, while Veillonella, Acidovorax, Kocuria and Delftia demonstrated increased abundance. To evaluate the generalizability of the model, we performed 10-fold cross-validation with stratified sampling. The cross-validated ROC curve yielded an AUC of 0.85 (95% CI: 0.627–0.943), with a sensitivity of 73.8% and specificity of 90.0% at the optimal cutoff determined by the Youden index (Figure 3D). These results suggest that the random forest model based on genus-level microbial abundances has good discriminatory ability for distinguishing DKD from controls.

Functional prediction of various gene sequences identified in the renal microbiome was conducted using the Kyoto Encyclopedia of Genes and Genomes (KEGG) database. Gene function analysis revealed distinct differences between the DKD and control groups in pathways related to environmental information processing and metabolism (Figure 3E). The DKD and control groups differed in functional pathways including cell motility, membrane transport, and transcription. Additionally, the control group showed greater enrichment in pathways related to endocrine and metabolic diseases. These results predict significant enrichment of microbial gene functions associated with specific material metabolism in the renal microbiome of DKD patients, suggesting potential microbe-related functional alterations in certain metabolic pathways.

Correlation analysis revealed associations between specific genera in the renal microbiome and key clinical parameters of DKD (Figure 3E). *Afipia, Acidovorax, Mesorhizobium*, and *Curvibacter* were positively correlated with fasting blood glucose (GLU) levels, while Ralstonia, Peptostreptococcus, and *Azospira* were negatively correlated with GLU levels. Niastella was positively correlated with eGFR, whereas *Rhodococcus* was negatively correlated with eGFR.

### 3.5 Altered renal tissue metabolite profiles in DKD patients versus controls

Untargeted metabolomics analysis was performed to identify altered metabolites in renal tissue samples from 20 DKD patients and 10 control subjects (Table 2). Following data normalization, 25,051 features were retained, including 1,094 superclass substances (Figure 4A). Multivariate statistical analyses, including Principal Component Analysis (PCA) and heatmap visualization, revealed distinct clustering and clear separation between DKD patients and control subjects (Figures 4B,C). In the DKD group, 461 metabolites were significantly downregulated and 186 metabolites were upregulated (Figure 4D).

**TABLE 2:** Clinical characteristics of the metabolomics cohort (‘x ± s).

Variables	control (*n* = 10)	DKD (*n* = 20)	*P*
eGFR (ml/min/1.73 m^2^)	77.04 ± 21.05	45.81 ± 23.47	0.001
H (cm)	168.55 ± 7.92	165.90 ± 9.72	0.430
W (kg)	69.85 ± 16.03	70.80 ± 14.78	0.873
BMI (kg/m^2^)	25.25 ± 4.38	24.75 ± 3.93	0.751
Age (years)	60.00 ± 11.87	45.85 ± 10.71	0.003
Scr (μmol/L)	79.20 (65.88, 95.60)	162.27 (129.30, 216.49)	< 0.001
BUN (mmol/L)	5.70 (4.30, 6.00)	11.05 (8.25, 18.02)	< 0.001
Hb (g/L)	138.00 (133.25, 142.25)	102.00 (89.00, 126.00)	0.003
Alb (g/L)	30.85 (28.15, 37.73)	38.35 (35.30, 42.32)	0.017
GLU (mmol/L)	5.51 (5.31, 5.96)	6.64 (5.23, 8.43)	0.307
Sex (Male)	4 (40.00)	15 (75.00)	0.108

Alb, serum albumin; Hb, hemoglobin; eGFR, estimated glomerular filtration rate; GLU, fasting blood glucose; BMI, body mass index; Cr, serum creatinine; SBP, systolic blood pressure; DBP, diastolic blood pressure.

**FIGURE 4:**
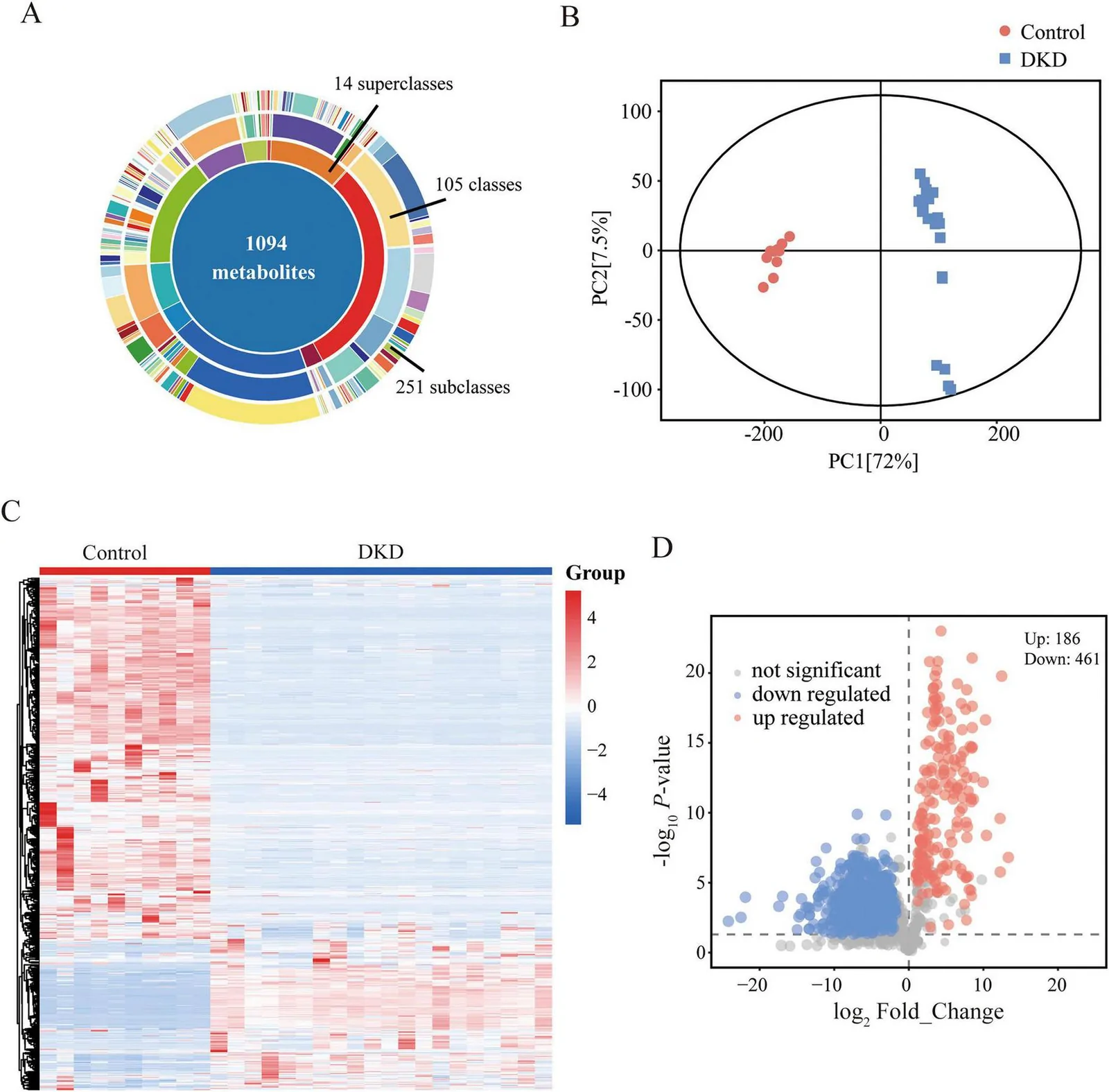
Changes of renal metabolites in DKD patients. **(A)** Circular plot of metabolite classification. The concentric rings represent different graded levels of metabolites, colors indicate distinct metabolite categories, and the area of each segment corresponds to the relative proportion of metabolites. **(B)** PCA score plot. The *x*-axis (PC1) and *y*-axis (PC2) represent the scores of the first and second principal components. Each point represents one sample, with color and shape indicating different groups. All samples fall within the 95% confidence interval. **(C)** Heatmap displaying metabolite profiles. The *x*-axis represents different groups, while the *y*-axis represents all metabolites. Colored tiles indicate relative expression levels of corresponding metabolites, with red indicating high expression and blue indicating low expression. **(D)** Volcano plot screening differential metabolites between groups. Each point represents a metabolite, with the *x*-axis showing log2-transformed fold changes and the *y*-axis showing -log10 transformed *P*-values from Student’s *t*-test.

### 3.6 Identification of core differential metabolites and functional prediction between DKD and control groups

Differential metabolites between the two groups were quantified by calculating the corresponding ratios to display metabolites with substantial changes. The top 5 significantly upregulated metabolites were pantothenol, 5-hydroxymethylfurfural, hexaethylene glycol, salicylic acid, gentisaldehyde. The top 5 significantly downregulated metabolites were valerylcarnitine, isovalerylcarnitine [Car (5:0)], palmitoyl sphingomyelin, butyrylcarnitine [Car (4:0)], isobutyrylcarnitine [Car (4:0)] (Figure 5A).

**FIGURE 5:**
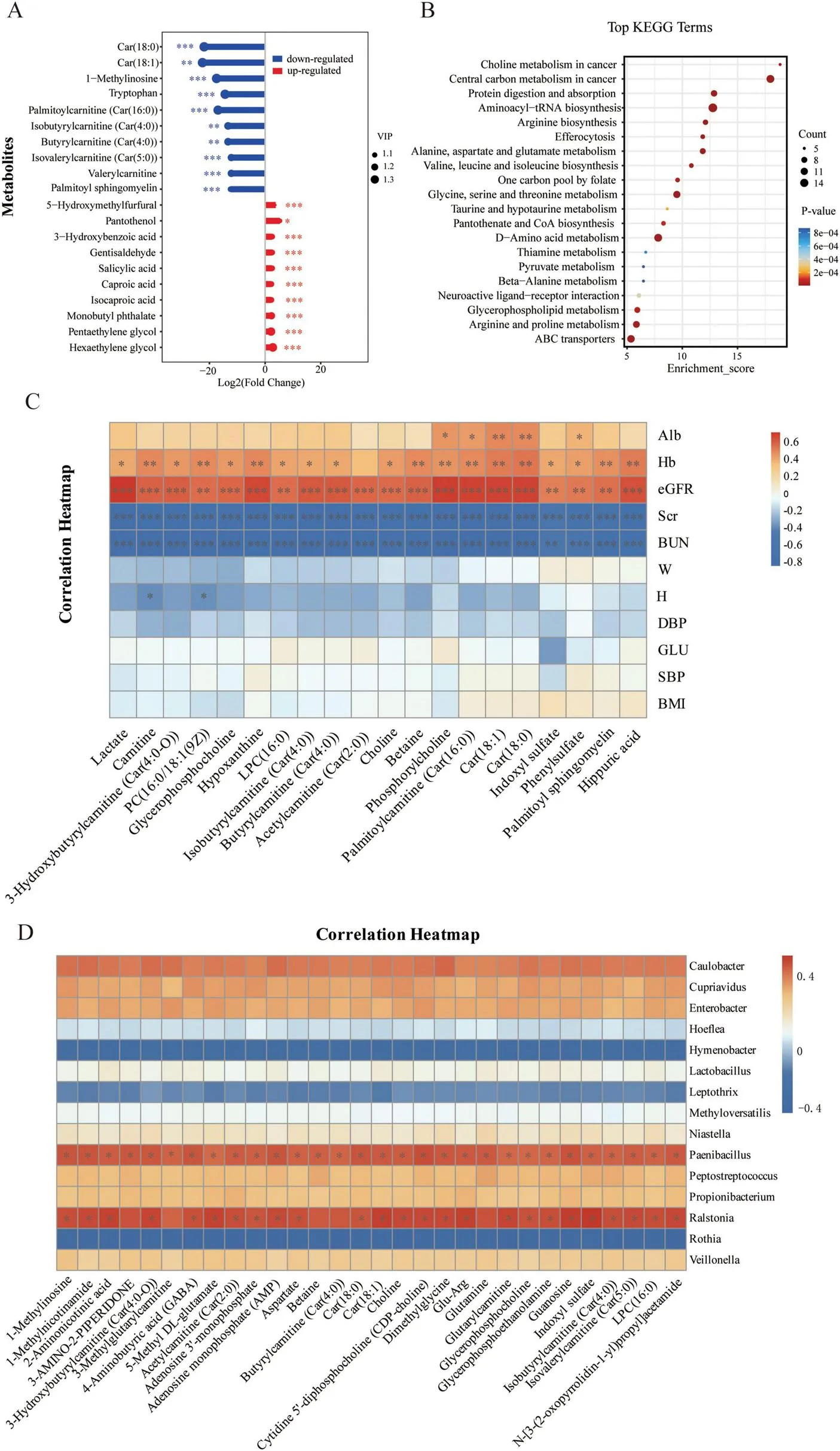
Characterization of differential metabolites in DKD patients. **(A)** Stick plot of Control group vs. DKD group. The *x*-axis shows logarithmically transformed fold changes. Point size represents VIP values from the OPLS-DA model, with larger points indicating higher VIP values. **(B)** KEGG enrichment plot of differential metabolites in DKD group versus control group. The *x*-axis indicates the differential abundance score for each pathway, and the *y*-axis shows KEGG metabolic pathway names. **(C)** Heatmap of correlations between metabolites and clinical features. **(D)** Heatmap of correlations between metabolites and renal microbiota. *p < 0.05; **p < 0.01; ***p < 0.001.

KEGG pathway annotation of core differential metabolites revealed significant alterations in specific pathways, primarily those involved in amino acid metabolism, including alanine, aspartate and glutamate metabolism, and valine, leucine and isoleucine biosynthesis (Figure 5B). Additionally, pathways related to organismal systems were also enriched. Correlation analysis between metabolite features and clinical characteristics in DKD patients showed that lactate, carnitine, 3-hydroxybutyrylcarnitine [Car(4:0-OH)] and other metabolites were positively correlated with eGFR, suggesting that specific alterations in renal metabolites are associated with established renal function impairment in DKD (Figure 5C). Meanwhile, correlation analysis of renal core microbiota showed that *Ralstonia* and *Paenibacillus* were significantly positively correlated with key metabolites such as 2-hydroxybutyrylcarnitine, glycerophosphocholine, oleylcarnitine and stearylcarnitine (Figure 5D).

### 3.7 Renal metabolites correlate with pathological features in DKD patients

All renal biopsy specimens were re-evaluated to assess pathological features, including mesangial matrix proliferation, Kimmelstiel-Wilson (K-W) nodule formation, tubular atrophy, and interstitial fibrosis in patients with DKD (Figures 6A,B). *Ralstonia* and *Bacillus* were positively correlated with the degree of fibrosis, whereas *Rhizobium* was negatively correlated with fibrosis. *Pseudomonas* and *Bosea* were positively correlated with the level of interstitial inflammation (Figure 6C).

**FIGURE 6:**
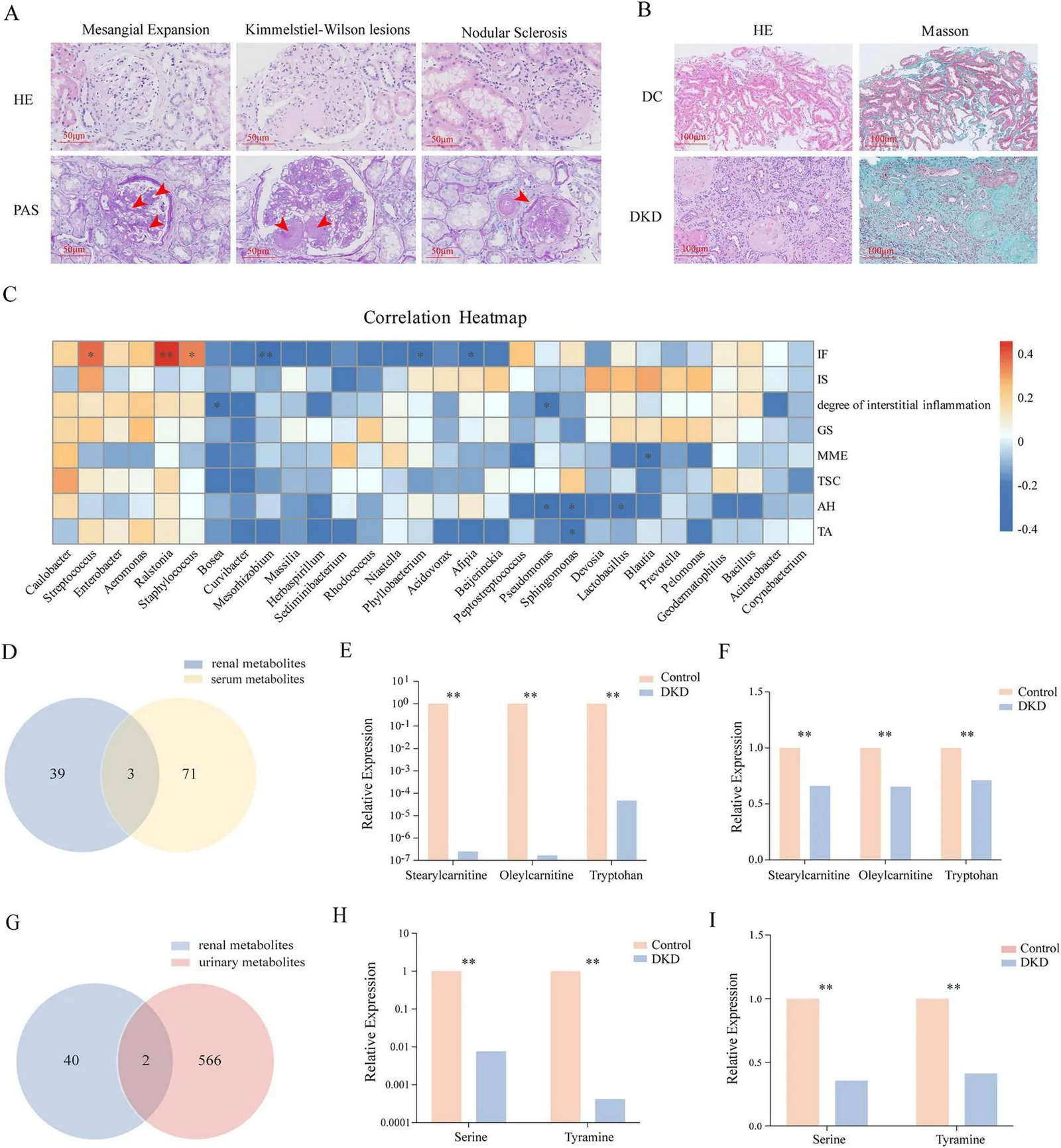
Correlation between differential metabolites and pathological features in DKD. **(A)** HE and PAS staining of renal tissue from a patient with diabetic nephropathy. Magnification: 400 × . **(B)** HE and Masson staining of renal tissue from control and diabetic nephropathy patients. Magnification: 200 × . **(C)** Heatmap of correlations between renal microbiome and histopathological characteristics. *p < 0.05; **p < 0.01; ***p < 0.001. **(D,G)** Venn diagram showing the screening of the union of metabolites. **(E,H)** Renal metabolome; **(F)** Serum metabolome; **(I)** Urinary metabolome. Change in metabolite levels between DKD and control groups. *p < 0.05; **p < 0.01; ***p < 0.001.

To assess the consistency of our findings with previously published DKD multi-omics data, we performed a literature-based comparison using external cohort data summarized in Supplementary Table 1. Comparison with external urinary and serum metabolomics datasets revealed that alterations in core renal metabolites were reflected in both biofluids (Figures 6D,G). Specifically, palmitoylcarnitine and stearylcarnitine were decreased in the renal metabolome of DKD patients (Figure 6E), consistent with reduced stearylcarnitine, oleylcarnitine, and tryptophan in the external serum metabolome (Figure 6F). Likewise, decreased renal serine and tyramine (Figure 6H) showed concordant reductions in the external urinary metabolome (Figure 6I). These cross-cohort comparisons should be interpreted as literature consistency rather than independent biomarker validation, as the external datasets were not re-analyzed through the same pipeline.

## 4 Discussion

In the present study, we characterized the microbial and metabolic profiles of renal tissue specimens from patients with DKD. Compared with serum metabolome, urine metabolome, urinary microbiome, and fecal microbiome analyses, renal tissue derived results reflect more specific and direct pathophysiological alteration in the renal tissue of DKD patients (Figure 7).

**FIGURE 7:**
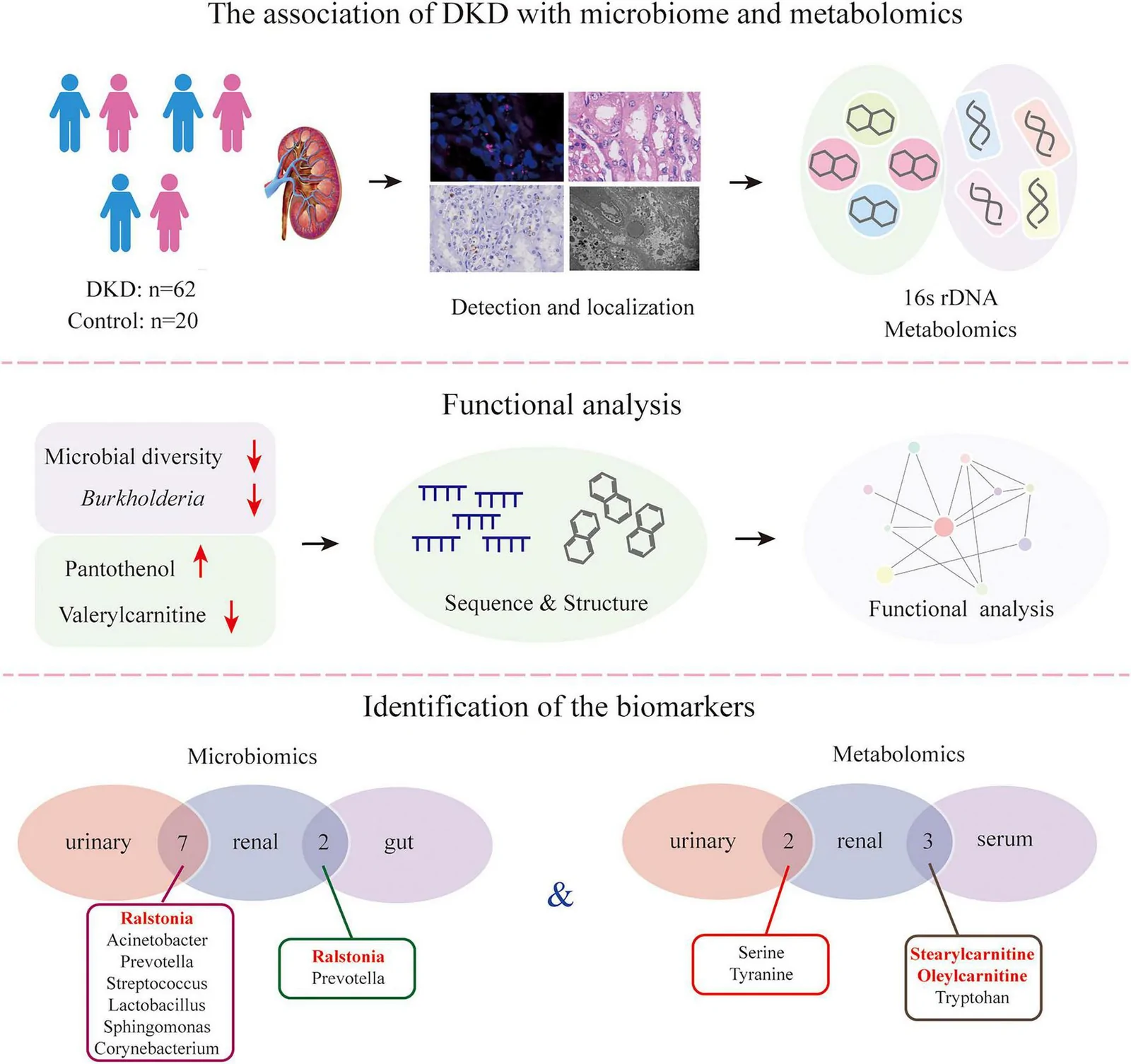
The association of DKD with microbiome and metabonomics. 16S rDNA sequencing and untargeted metabolomics of renal tissue from DKD patients reveal altered microbial and metabolic diversity, functional shifts, and candidate biomarkers.

As reported by Linh et.al and Liu et.al, viable bacteria exist in renal tissues of both in mice and humans, as demonstrated using EUB338 probe (Linh et al., 2022; Liu et al., 2022). In this study, we provided further evidence for the presence of intrarenal bacteria in both DKD patients and controls using FISH, IHC and TEM (Nejman et al., 2020). We subsequently performed renal tissue microbiome and metabolome profiling to investigate compositional and functional differences in intrarenal bacteria and metabolites between DKD patients and controls. Consistent with previous findings from urinary and fecal microbiome studies, the DKD group exhibited no significant reduction in alpha diversity but a significant difference in beta diversity compared with controls (Yang et al., 2023; Li et al., 2025). The diminished diversity of the renal microbiome suggests that DKD progression is associated with shifts in the composition and abundance of intrarenal bacteria, which may be closely linked to the deterioration of renal function.

Among the top 30 urinary microbiota species, 7 overlapped with those in the renal microbiota, and an additional 5 species were also detectable among the non-top 30 renal taxa19. In contrast, only 3 species from the top 30 gut microbiota overlap with the top 30 renal species, with only 2 further species identifiable in the non-top 30 gut taxa (Wu et al., 2025). Together, these results demonstrate that renal tissue microbiota is more closely related to the urinary microbiota than to the gut microbiota.

*Ralstonia* is a genus of opportunistic pathogens associated with a wide spectrum of infections, including meningitis and spherical pneumonia (Liao et al., 2023; Ma et al., 2023). In our study, *Ralstonia* was the most abundant bacterial genus in renal tissues of DKD patients, yet its abundance was significantly lower in the DKD group than in controls. This seemingly paradoxical finding—reduced abundance but positive correlation with interstitial fibrosis—can be understood through the lens of microbial community network dynamics. Recent studies have demonstrated that the microbial community constitutes an intricate interactive network, rather than a simple collection of individual microbes with one-to-one disease associations. In healthy controls, the intact interactive balance of renal microbiota constrains the pathogenic potential of *Ralstonia*, even though its abundance is relatively high. In contrast, structural disruption of the renal microbial community under diabetic kidney disease removes such inhibitory interspecies interactions, unmasking the pathogenic effects of *Ralstonia*. This framework may explains the positive correlation between its abundance and the severity of interstitial fibrosis observed in DKD patients. Furthermore, because our 16S sequencing data reflect relative rather than absolute abundance, the decreased relative abundance of *Ralstonia* in DKD may be attributed to overall compositional restructuring of the microbial community, rather than an absolute depletion of this genus.

We further compared the abundance changes of *Ralstonia* in the urinary microbiome with those observed in our renal tissue dataset (Li et al., 2025). Consistent with our findings, a previous study reported reduced renal *Ralstonia* abundance in injured db/db mice compared with wild-type controls (Lu et al., 2025). Based on the Mean Decrease Accuracy metric, *Ralstonia* and other key microorganisms were identified as the most important discriminatory features in the classification model. The random forest model further emphasized the ability of these microbial taxa to distinguish DKD patients from controls, suggesting a profound impact of DKD on the structure of the renal microbial community. Notably, *Ralstonia* abundance was negatively correlated with blood glucose (R = 0.42, *p* = 0.002) and BUN (R = 0.30, *p* = 0.028). Collectively, these findings suggest *Ralstonia* as a candidate diagnostic indicator for DKD.

Emerging evidence suggests that renal bacteria may originate via translocation from the gut microbiota (Linh et al., 2022; Liu et al., 2022). Interestingly, previous studies reported increased fecal *Ralstonia* abundance in DKD patients (Hu et al., 2025). This discrepancy may be explained by impaired translocation and reduced renal retention of *Ralstonia* in DKD, accompanied by enhanced fecal excretion.

Serine has been identified as a differential metabolite in urinary metabolomic studies comparing DKD patients with healthy controls (Jiang et al., 2023). Sun et al. demonstrated that combining machine learning approaches with urinary metabolomics led to the identification of five differentially expressed metabolites: hypoxanthine, N-acetyl-DL-histidine, cortisol, tetrahydrobiopterin and L-kynurenine (Sun et al., 2025). Plasma biomarkers associated with DKD progression include branched-chain amino acids (BCAA), creatinine, N,N,N-trimethyl-L-alanyl-L-prolinebetaine (TMAP), urea, spermine, asymmetric dimethylarginine, imidazolepropionic acid, adipoylcarnitine and 1-methylhistidine (Gurung et al., 2025; Hu et al., 2025; Rana et al., 2025). The discrepancies in these biomarkers across different studies are attributed not only to variations in patient cohorts but also to the fact that urinary and serum metabolites reflect systemic metabolic changes rather than renal-specific alterations. These inconsistencies further exacerbate the heterogeneity of DKD, making it challenging to accurately identify DKD-specific biomarkers. Therefore, the present study characterized renal metabolic changes in DKD patients using renal tissue specimens, which may provide specifically relevant candidate markers associated with DKD pathogenesis. However, renal tissue biopsy is an invasive procedure with inherent challenges in specimen collection. Consequently, this study integrated serum metabolomics and urinary metabolomics to identify biomarkers that not only accurately reflect renal injury but also enable convenient and non-invasive detection in clinical setting. Few studies have reported stearylcarnitine, oleylcarnitine, tryptophan, serine and tyramine as potential biomarkers, despite their significantly altered level in DKD. In the present study, stearylcarnitine and oleylcarnitine exhibited consistent reductions in both serum and renal tissue. Moreover, their levels were positively correlated with eGFR and negatively correlated with serum creatinine, two key indicators of renal function. Taken together, these findings suggest that serum stearylcarnitine and oleylcarnitine may serve as promising biomarkers reflecting the renal dysfunction in DKD.

In conclusion, our study may be the first to explore renal microbiota and metabolism in DKD. Stearylcarnitine and oleylcarnitine may be the promising biomarker in serum that can help distinguish the renal dysfunction in DKD. However, we need more studies to confirm this in the future.

## Data Availability

The datasets presented in this study can be found in online repositories. The names of the repository/repositories and accession number(s) can be found below: GSA: CRA044671 (https://ngdc.cncb.ac.cn/gsa/s/zNyy6166).

## Supplementary material

Supplementary Figure 1 The study of flowchart.
