# Supplementary material for: Renal tissue microbiota and metabolite profiling reveal dysregulated signatures in diabetic kidney disease
Source: Front Microbiol. 2026 Sep 17;17:1898177. doi: 10.3389/fmicb.2026.1898177 (PMC13628504; doi:10.3389/fmicb.2026.1898177)
Supplement: Supplementary file 1 [file Data_Sheet_1.docx]

Figure S1

Figure S1 The control of FISH

Figure S2

Figure S2 The study of flowchart

Figure S3

Figure S3 Sample Control for Metabolomics

Figure S3 Sample Control for Metabolomics. A, Positive-mode EIC plots of internal standards from QC samples; B, Negative-mode EIC plots of internal standards from QC samples; C, Positive-mode EIC plots of internal standards from blank and QC samples; D, Negative-mode EIC plots of internal standards from blank and QC samples; E, PCA score plot; F, Correlation analysis of QC samples; G, Stability of internal standard responses in QC samples

Figure S 4

Figure S4 PCA score plot with QC

Figure S5

Figure S5 Correlation heatmaps of microbial genera and metabolites with clinical and pathological features in DKD. A, Top 10 differential species between DKD and control groups.B, Heatmap of microbial genera vs. clinical features in DKD. C, Heatmap of microbial genera vs. pathological features in DKD. D, Heatmap of metabolites genera vs. pathological features in DKD.E, Heatmap of metabolites genera vs. clinical. features in DKD. **: p < 0.05; **: p < 0.01; ***: p < 0.001.; # q < 0.05, ## q < 0.01, ### q < 0.001* (FDR-adjusted q-values).

Table S1

Table S1 Summary of external datasets

|  | **gut microbiome** | **urinary microbiome** | **serum metabolites** | **urinary metabolites** |
| --- | --- | --- | --- | --- |
| **Dataset source** | 10.1038/s41598-025-91281-9 | 10.1186/s12967-025-06405-6 | 10.1038/s41598-025-91281-9 | 10.1007/s11255-022-03326-x |
| **Sample size** | 90 subjects were recruited: 30 with DKD, 30 with type 2 diabetes mellitus (T2DM), and 30 normal controls (NCs) | Healthy controls, patients with DKD with microalbuminuria (DKD1 group) or macroalbuminuria (DKD2 group), and patients with T2DM were recruited (n = 20 for each group). | 90 subjects were recruited: 30 with DKD, 30 with type 2 diabetes mellitus (T2DM), and 30 normal controls (NCs) | 79 participants, including healthy subjects (n = 20), T2DM patients (n = 20), 39 DKD patients |
| **Platform/method** | 16S rRNA sequencing | 2bRAD sequencing for microbiome (2bRAD-M) | untargeted metabolomics | liquid chromatography-mass spectrometry-based metabolomics method |
